# Supplementary material for: Ha-RasV12-Induced Multilayer Cellular Aggregates Is Mediated by Rac1 Activation Rather Than YAP Activation
Source: Biomedicines. 2022 Apr 23;10(5):977. doi: 10.3390/biomedicines10050977 (PMC9138672; doi:10.3390/biomedicines10050977)
Supplement: Supplementary file 1 [file biomedicines-10-00977-s001.zip › biomedicines-1621881-supplementary.pdf]

biomedicines

MDPI

Supplement

S1. Figures, Tables and Schemes

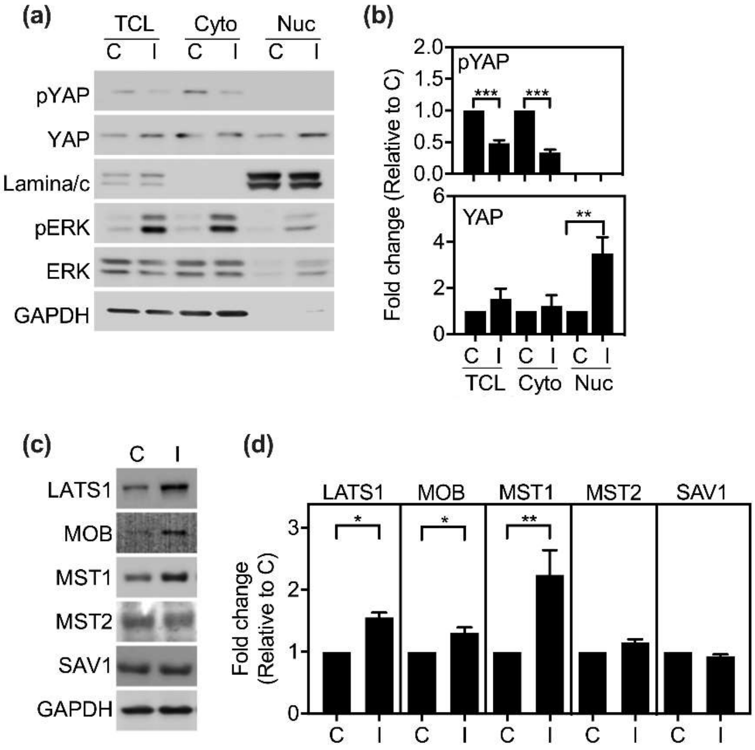

Figure S1. Ha-Ras^{V12}-induced YAP nuclear translocation through Hippo-independent pathway.

MK4 cells at overconfluent stage were treated with or without 5 mM IPTG for 24 hr. (**a**) Representative immunoblots of cellular fractions of cells. The protein levels of pYAP (S127), YAP, pERK and ERK were analyzed. GAPDH was served as internal control and cytoplasmic marker. Lamin A/C was served as nuclear marker. (**b**) Quantification results of pYAP and YAP from (**a**). (**c**) Representative immunoblots of hippo pathway associated protein, including LATS1, MOB, MST1, MST2 and SAV1. GAPDH was served as internal control. (**d**) Quantification results of hippo pathway associated protein form (**c**). All data are expressed as the mean ± SEM from three independent experiments. * p <0.05, ** p <0.01, *** p < 0.001.

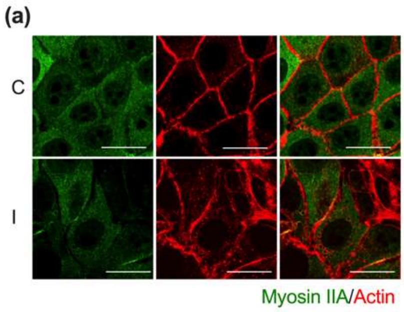

Figure S2. The distribution of myosin IIA did not change at confluent stage after Ha-Ras^{V12} overexpression.

MK4 cells at overconfluent stage were treated with or without IPTG (5 mM) for 24 h. (**a**) Representative confocal images of cells were stained with myosin IIA (green) and Phalloidin (red). Scale Bar = 20 µm.

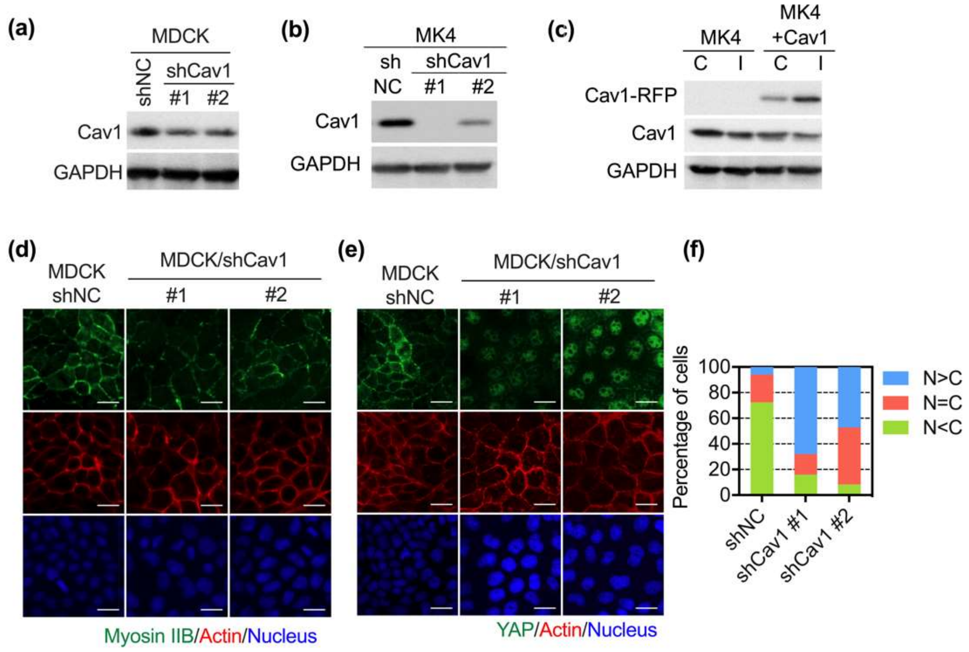

Figure S3. Absence of Cav1 inhibited junctional myosin IIB and induced YAP nuclear translocation in MDCK cells.

(**a**) and (**b**) Representative immunoblots result of Cav1 to indicated the efficiency of Cav1 knockdown in MDCK and MK4 cells. GAPDH was served as internal control. (**c**) Representative immunoblots result of Cav1 to indicated the efficiency of Cav1 overexpression MK4 cells. GAPDH was served as internal control. Knockdown of Cav1 in MDCK cells were cultured at overconfluent stage. (**d**) Representative immunofluorescence images of cells at confluent stage were stained with myosin IIB (green), Phalloidin (red), and Hoechst 33258 (blue). Scale bar = 20 μm. (**e**) Representative immunofluorescence images of cells at confluent stage were stained with YAP (green), Phalloidin (red), and Hoechst 33258 (blue). Scale bar = 20 μm. (**f**) Percentage of cells with predominant nuclear YAP localization (N>C), equal nuclear and cytosol YAP localization (N=C), and predominant cytosol YAP localization (N<C).

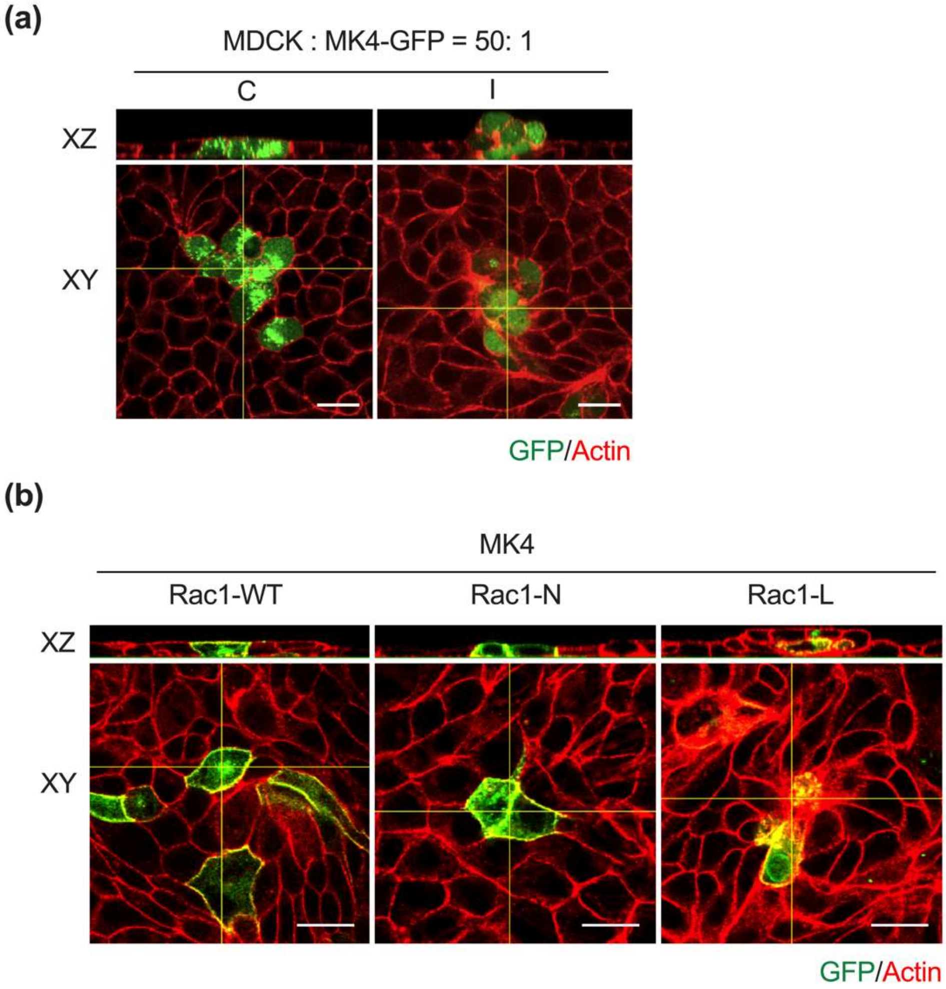

Figure S4. Activation of Rac is required for multilayer cellular aggregates.

MDCK cells were co-culture with MK4 overexpressed GFP cells in the ratio of 50:1. Cells were treated with or without IPTG(5mM) for further 24 hr. (**a**) Representative confocal images and xz cross-sections images showed MK4-GFP and, cells were stained with Phalloidin (red). Scale Bar = 10 µm. MK4 cells were transfected with GFP-conjugated Rac1-WT, Rac1-L and Rac1-N transiently. (**b**) Representative confocal images and xz cross-sections images showed Rac1-GFP (green) and, cells were stained with Phalloidin (red). Scale bar = 20 µm.

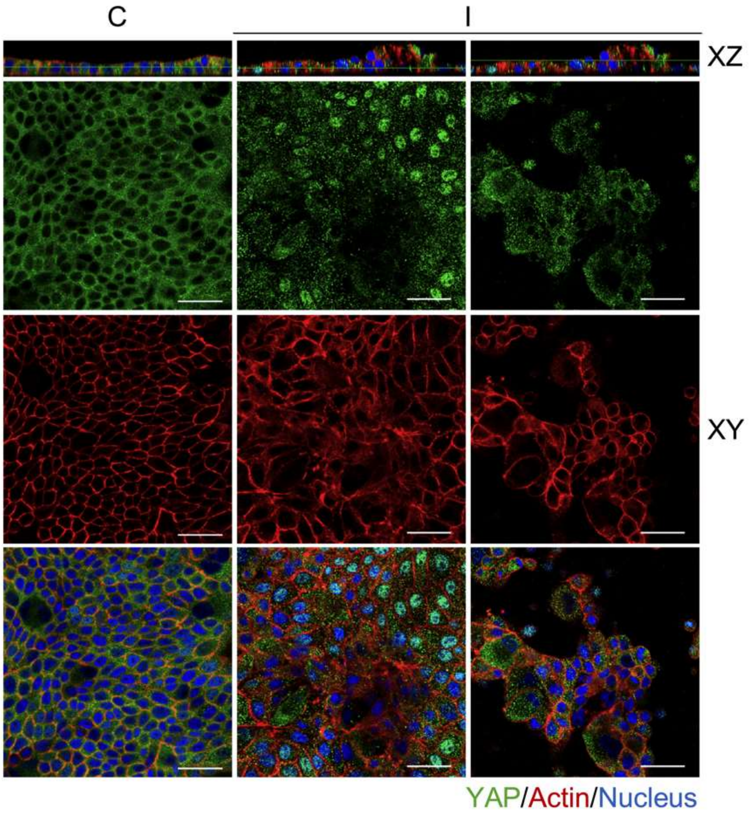

Figure S5. YAP translocated to cytosol in multilayer cellular aggregates area in Ha-Ras^{V12}-overexpressing cells.

MK4 cells were treated with or without IPTG (5mM) for 24 hr. Representative confocal images and xz cross-sections images of cells were stained with YAP (green), Phalloidin (red), and Hoechst 33258 (blue). The green line in xz cross-sections images indicated the section of xy cross-sections images. Scale bar = 40 μm.
